# Datasets on the spatial distribution of mercury and its controlling factors in the Yellow Sea

**DOI:** 10.1016/j.dib.2021.106792

**Published:** 2021-01-26

**Authors:** Do Hyun Jeong, Wooyoung Jeong, Saehun Baeg, Jihun Kim

**Affiliations:** a South Sea Research Institute, Korea Institute of Ocean Science & Technology, Geoje 53201, Republic of Korea; b KIOST School, University of Science & Technology, Daejeon 34113, Republic Korea

**Keywords:** Total mercury, Surface sediments, Spatial distribution, Controlling factors, Yellow Sea and the northern East China Sea

## Abstract

Large amount of anthropogenic mercury (Hg) emitted from China has been transported and deposited in the northwestern Pacific marginal seas; in particular, the Yellow Sea adjacent to China is immediately affected by Chinese-high Hg emissions [1,2]. This article presents the comprehensive baseline dataset on the mercury concentrations and their controlling factors in surface sediments from the entire Yellow Sea shelf, including Korean and Chinese rivers and coastal zones. These data supported the research article entitled “Sedimentary mercury (Hg) in the marginal seas adjacent to Chinese High-Hg emissions: source-to-sink, mass inventory, and accumulation history” Kim et al. [1]. Some of the data was used in Kim et al.’s research paper [3] with the reference [1]. A total of 492 surface sediments were collected from the Yellow Sea shelf and coastal zones, and the rivers around the Sea. All sediment samples were freeze-dried and ground by agate mortar for analyzing total mercury (THg) and related elemental components (total nitrogen, total carbon, total inorganic carbon, total organic carbon, and aluminum). Most previous studies on the sedimentary Hg were conducted locally, mainly in the river-dominated coastal and inner shelf zones of the Yellow Sea, which are associated with riverine Hg inputs. Thus, the quality and quantity of available sedimentary Hg data, on which we rely for mass inventories of Hg in the Sea, are limited. In this respect, our large dataset may contribute significantly to a better understanding of the behaviors of riverine and atmospheric Hg from Chinese sources and will help to further refine global estimates of Hg discharge to ocean margins and open oceans in East Asia. Additionally, the dataset will be essential for improving numerical model for global budget calculation and prediction.

**Specifications Table**

**Table:** 

Subject	Earth and Planetary Sciences
Specific subject area	Geochemistry, Sediment pollution
Type of data	Figures, Tables
How data were acquired	Sediment sampling: grab sampler and box corer; Total nitrogen (TN) and total carbon (TC) contents: CHN elemental analyzer (FLASH 2000, Thermo Fisher Scientific, USA); Total inorganic carbon (TIC) contents: CO_2_ coulometer (CM5014, UIC, USA); Total mercury (THg) concentrations: Automatic Hg analyzer (model Hydra II C Direct Hg analyzer; Teledyne Leeman Labs, Hudson, NH, USA); Aluminium (Al) concentrations: Inductively coupled plasma atomic emission spectroscopy (ICP-AES; Spectro Flame Modula EOP; SPECTRO Analytical Instruments Inc., Germany)
Data format	Raw data, Analyzed
Parameters for data collection	Field collection of riverine, coastal, and shelf sediment samples covering the entire Yellow Sea. Determination of THg, and related element components (TN, TC, TIC, TOC, and Al).
Description of data collection	A total of 492 surface sediment samples were collected by grab sampler or box corer between 2001 and 2010 to achieve extensive spatial coverage of the entire Yellow Sea. The sediment samples were collected at a depth of approximately 1 cm. Loss of the top of the sample due to the use of a grab sampler was likely minimal due to the fine-grained cohesive aggregate compositions that are prevalent in these basins. The concentrations of THg and related elements (TN, TIC, TIC, TOC, Al) were analyzed for these riverine, coastal, and shelf sediments, together with reference materials. Total organic carbon (TOC) contents were calculated from the difference between TC and TIC contents. A large dataset of THg-Al-TOC contents was established for a total of 492 surface sediment samples covering the entire Yellow Sea.
Data source location	Yellow Sea, the northwest Pacific margin [30∼38°N, 117∼129°E]
Data accessibility	With the article
Related research article	Jihun Kim, Dhongil Lim, Do hyun Jung, Jeongwon Kang, Hoisoo Jung, Hanjun Woo, Kapsik Jeong, Zhaokai Xu, Sedimentary mercury (Hg) in the marginal seas adjacent to Chinese high-Hg emissions: Source-to-sink, mass inventory, and accumulation history, Marine Pollution Bulletin. 128 (2018) 428-437. https://doi.org/10.1016/j.marpolbul.2018.01.058

**Value of the Data**

• In the entire Yellow Sea, the quality and quantity of available sedimentary Hg data are highly limited because most previous studies on the sedimentary Hg were conducted locally, mainly in the coastal zones of the Sea. Because of this, our understanding of source-to-sink, flux, budget, and mass inventory of sedimentary Hg for the entire Yellow Sea is not yet sufficient.
• This is the large comprehensive dataset for sedimentary Hg and related elemental components for a total of 492 surface sediment samples covering the entire Yellow Sea.
• This baseline dataset of THg-TOC-Al in the Yellow Sea will be invaluable for the researchers studying the global Hg inventory (especially, numerical modeling study) as it yields crucial and essential information for calculating the quantitative contribution (i.e., depositional flux and budget) of Chinese-emitted Hg in the northwestern Pacific marginal seas as well as other open oceans.
• Our dataset can also be used to accurately estimate the natural background level of Hg to better assess the anthropogenic Hg impacts on marine environments and ecosystems in the marginal seas.

## 1 Data Description

**Fig. 1:**
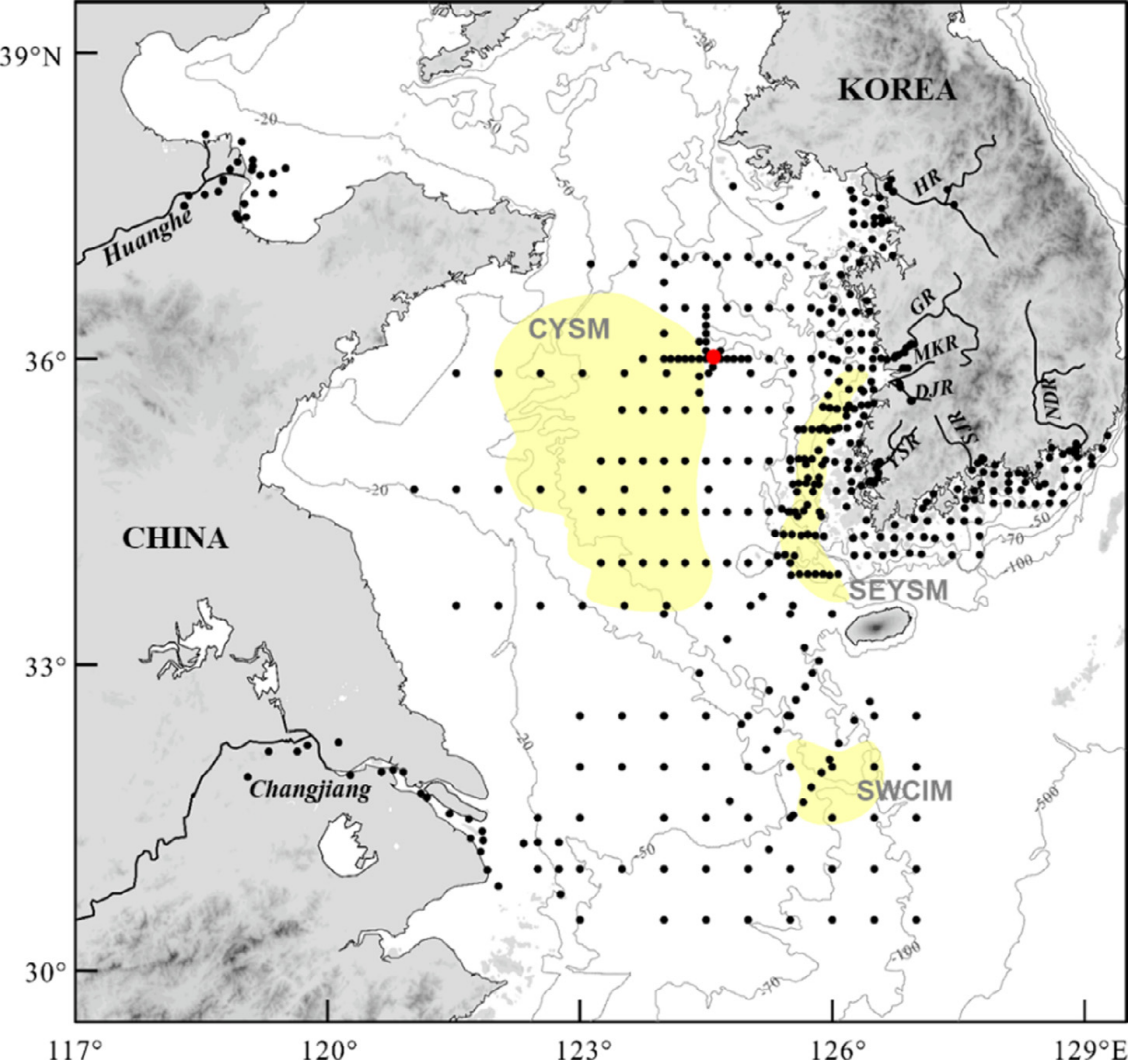
Bathymetric map of the study area showing the 492 sampling sites (dots) and three shelf mud patches (shaded). Red circle: ocean waste-disposal site. CYSM: the central Yellow Sea mud zone, SEYSM: the southeastern Yellow Sea mud zone, SWCIM: the southwestern Cheju Island mud zone, HR: Han River, GR: Geum River, MKR: Mankyeong River, DJR: Dongjin Rivers, YSR: Yeongsan River, SJR: Seomjin River, NDR: Nakdong River.

A total of 492 surface sediments were collected by Smith-Mcintyre or van Veen grab sampler and box corer in the entire Yellow Sea shelf, including Korean (Han River, Geum River, Yeongsan River, Mankyeong River, Dongjing River, Seomjin River, Nakdong River) and Chinese (Huanghe and Changjiang) rivers, and coastal zones (Fig. 1). The original dataset for THg and related elemental components (TN, TC, TIC, TOC, and Al), including site locations, and their statistic summary are presented in Table S1 and Table 1, respectively.

**Table 1:** Statistic summary of concentration of total mercury (THg) and related elemental components in the surface sediments of the Yellow Sea, including Korean and Chinese rivers.

	N	Average	Standard deviation	Minimum	Maximum
TN (%)	492	0.07	0.05	0.00	0.32
TC (%)	492	1.17	1.04	0.04	12.15
TIC (%)	492	0.59	1.01	0.00	11.99
TOC (%)	492	0.57	0.42	0.02	2.66
Al (%)	385	6.4	1.5	1.6	10.4
Hg (ng/g)	492	25.8	27.9	1.1	215.7

**Fig. 2:**
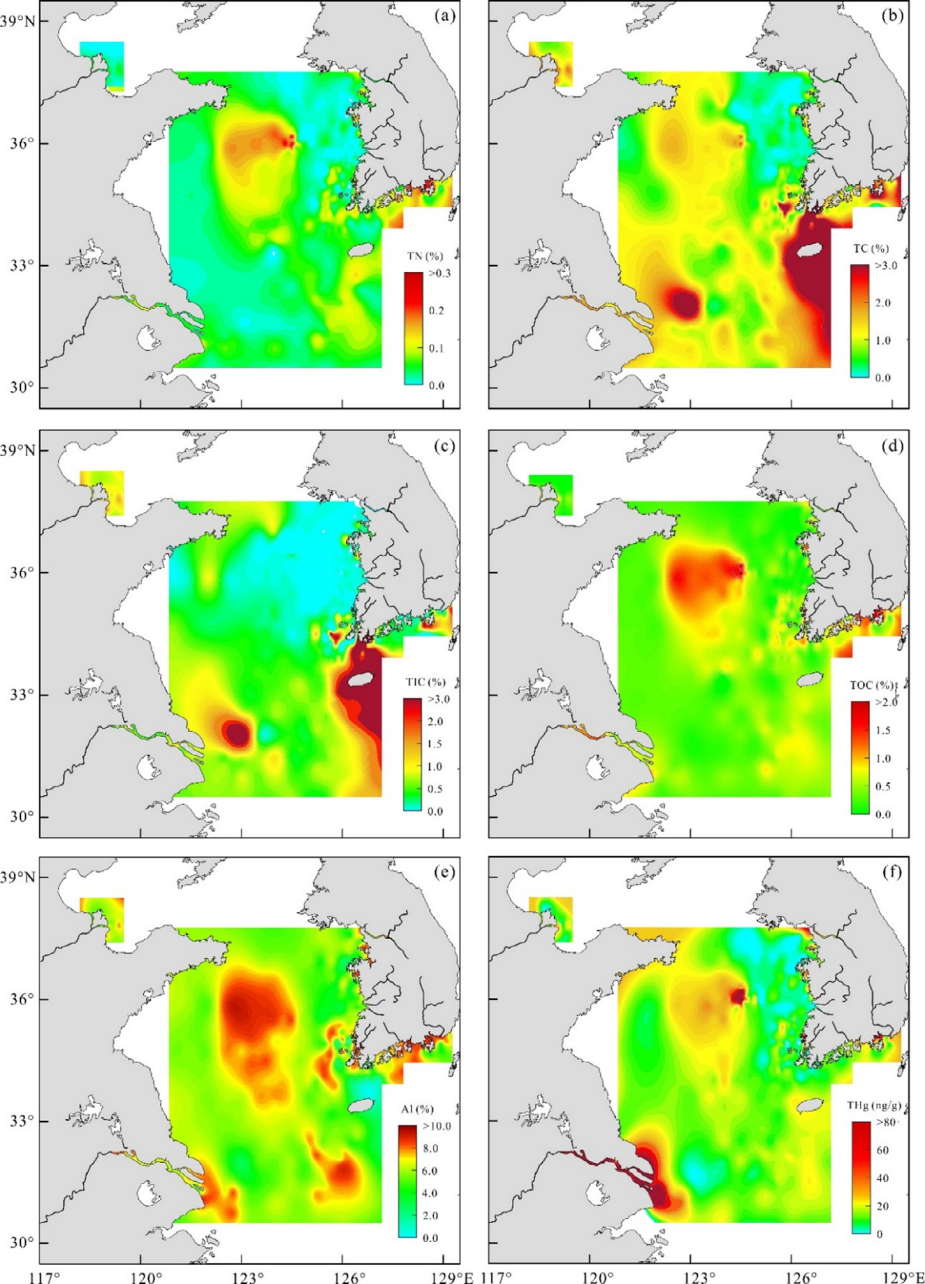
Spatial distribution of concentrations of (a) total nitrogen, (b) total carbon, (c) total inorganic carbon, (d) total organic carbon, and (e) Al concentrations (f) total mercury in the Yellow Sea.

TN and TC contents vary from 0.00 to 0.32% (average 0.07±0.05%) and 0.04 to 12.15% (average 1.17±1.04%), respectively. Overall, TN contents are the highest (> 0.15%) in the ocean waste-disposal site of the central Yellow Sea and the southern Korean coastal zone, followed by three shelf mud zones (CYSM, SEYSM, and SWCIM, Fig. 1) with a range of 0.08–0.15% (Fig. 2a). These shelf mud deposits consist mostly of fine-grained muds and silty muds (> 76 phi in mean grain size, < 10% in sand contents) [4]. TC contents also are relatively higher (> 2.5%) in sediments of the southern Korean coasts and around the SWCIM region, followed by Hunanghe and Changjiang sediments (1.5–2.5%) (Fig. 2b). TIC contents vary between 0.00 and 11.99% (average 0.59±1.01%) (Fig. 2c): overall, the contents are higher in Hunahge sediments (> 1%) and SWCIM sediments (> 1.5%), followed by Changjiang sediments. TOC contents [TOC (%) = TC (%) − TIC (%)] fluctuate between 0.02 and 2.55% (average 0.57±0.42%), having a spatial distribution similar to TN (Fig. 2d). TOC is higher than 1% in sediments of Changjiang and coastal zone and ocean waste-disposal site, followed by three fine-grained shelf mud deposits (0.5–1%). Excluding these sediments, most sediments are less than 0.5% in contents.

Surface sediments of the study area exhibit a broad variation in Al concentration, ranging from 1.6% to 10.4% (average 6.4 ± 1.5%), with a relatively high level of Al (> 7%) in three fine-grained shelf muddy sediments, and coastal and riverine sediments (Fig. 2e). In general, Al concentration highly dependent on grain size: Al concentration increases as the contents of fine-grained particles in the sediments increase. As shown in Fig. 2, Al shows a similar spatial distribution pattern with TOC and TN, showing these elements are constrained primarily by the sediment grain size.

THg concentrations in the study area ranged from 1.1 to 215.7 ng/g (average 25.8 ± 27.9 ng/g) with significant spatial variation (Fig. 2f). Unlike to Al concentration, the highest concentrations are observed in the rivers (Korean rivers: 12.2–134.6 ng/g; Changjiang: 56.5–215.7 ng/g; Huanghe: 7.9–71.0 ng/g) and their estuarine and coastal zones (> 50 ng/g). Characteristically, the concentrations in the ocean waste-disposal site (23.4–145.0 ng/g) are as high as those in the coastal sediments, followed by the shelf mud zones (25–40 ng/g) of the central Yellow Sea. Except for these areas, most of the shelf sediments are less than 20 ng/g in concentration. Overall, the THg concentrations in the rivers and their coastal zones, and the ocean waste-disposal site are higher compared to the upper continental crust (50 ng/g, [5]) and the natural values (< 50 ng/g, [6]). In the Yellow Sea shelf area, the concentration is relatively lower than in the East/Japan Sea (30–164 ng/g), Okhotsk Basin (36–55 ng/g), and Western Arctic Ocean (62–68 ng/g) [3].

## 2 Experimental Design, Materials and Methods

East Asia (mainly China and India) is one of the largest Hg emission source regions in the world; for example, China contributes about 30 and 50% of global and Asian anthropogenic Hg emissions, respectively [7,8]. In this respect, the study of sedimentary Hg in Chinese marginal seas (e.g., Yellow Sea, East China Sea, and East/Japan Sea) may yield valuable information about regional and global Hg cycles as well as the behavior of anthropogenic Hg. In particular, the Yellow Sea might be vulnerable to the impact of anthropogenic Hg, and their sediments are probably important reservoirs for Chinese land-based Hg via atmospheric and riverine transport. However, there is no available sedimentary Hg dataset covering the entire Yellow Sea because many works of sedimentary Hg are restricted to the local areas, such as estuarine and coastal areas, and severely polluted bays [e.g., [9], [10], [11]]. In addition, such limited availability of data may provide relatively rough calculations and predictions in the numerical model. Thus, a large comprehensive dataset of sedimentary Hg with its controlling components in the entire Yellow Sea has been required for better understanding the behaviors of riverine and atmospheric Hg from Chinese sources.

To achieve large THg data, a total of 492 surface sediment samples were collected in the entire Yellow Sea shelf, including Korean and Chinese rivers, estuaries, and coastal zones (Fig. 1). Simultaneously, data on controlling factors affecting Hg deposition were gathered; it is well known that organic-enriched fine particles likely accelerate the deposition of Hg to the sediments through a scavenging process in the water column and consequently control the Hg distribution in the sediments [12,13]. Thus, a comprehensive THg-Al-TOC dataset (including TN and TIC) for 492 surface sediment samples covering the entire Yellow Sea has been establised.

Surface sediment samples were collected by grab sampler and box corer between 2001 and 2010 to achieve extensive spatial coverage of the entire Yellow Sea. All sediment samples were stored in an onboard freezer, then were freeze-dried for 1–2 days to be ground to a powder with a mortar and pestle (model pulverisette 6, FRITSCH, Germany). About 50 mg of powdered sample was analyzed for THg concentrations using an automatic Hg analyzer, involved thermal decomposition followed by catalytic reduction, amalgamation, desorption, and the use of an atomic absorption module (model Hydra C II Direct Hg analyzer; Teledyne Leeman Labs, Hudson, NH, USA), which is based on US EPA 7473 [14]. The powdered sample in the nickel boat is pyrolyzed at about 850 °C and oxidized Hg is transformed into elemental mercury (Hg^0^) through a catalyst tube. Then, Hg^0^ gasses are detected using 820 nm wavelength. THg concentrations were calibrated against marine sediment reference material (MESS-3; total Hg=91±9 ng/g certified by the National Research Council of Canada) for trace metals and other constituents. The detection limit was obtained less than 0.02 ng. In measurements of the Hg concentration, the analytical accuracy was determined to be < 5%, based on replicate analysis of the certified reference material (MESS-3). The analytical precision was determined to be < 10% based on replicate measurements of standard materials and sediment samples.

For TN and TC contents, the about 15 mg powdered sediment samples with reference material (L-cystine; 11.66% N, 29.99% C) were analyzed using a CHN elemental analyzer (FLASH 2000, Thermo Fisher Scientific, USA). The encapsulated powder samples by tin are combusted in silvered cobaltous-cobaltic oxide and chromium oxide filled tube at 900 °C then reduced in copper oxide filled tube at 550 °C. On the basis of replicated measurements of a standard material and sediment samples, the accuracy and precision in TN and TC measurements were within 5%. TIC contents were measured using a CO_2_ coulometer (CM5014, UIC, USA) by reacting approximately 15 mg powdered samples with 40% perchloric acid. The precision and accuracy in TIC analyses were < 5% in replicated reference material (calcium carbonate having 12% C) and sediment samples measurements. TOC contents were calculated by subtracting TIC from TC contents.

For Al concentrations, about 0.2 g powdered samples, including an international standard reference material (MAG-1; Al_2_O_3_=16.4, 0.3%; certified by U.S Geological Survey), were dissolved in a mixture of hydrofluoric-perchloric-nitric acids in an airtight Teflon bomb. These solutions were then analyzed to determine Al concentration using inductively coupled plasma atomic emission spectroscopy (ICP-AES, model Spectro Flame Modula EOP; SPECTRO Analytical Instruments Inc., Germany). The precision and accuracy of concentrations were monitored by repeated analyses of the standard MAG-1. Differences between the determined and recommended values were less than 5%, and the leaching efficiency checked by MAG-1 was about 90%.

## CRediT Author Statement

**Do Hyun Jeong:** Resources, Formal analysis, Validation, Data Curation, Writing-Original Draft; **Wooyoung Jeong:** Investigation, Resources, Formal analysis; **Saehun Baeg:** Investigation, Resources, Formal analysis; **Jihun Kim:** Conceptualization, Validation, Writing-Original Draft, Writing-Review & Editing, Visualization, Supervision and Data analysis.

## Declaration of Competing Interest

The authors declare that they have no known competing financial interests or personal relationships which have, or could be perceived to have, influenced the work reported in this article.

## Appendix Supplementary materials

Image, application 1
